# Cardiovascular Risk Profiles of Individuals with Bruxism: A Case-Control Study Using QRISK3 Scores

**DOI:** 10.7759/cureus.93155

**Published:** 2025-09-24

**Authors:** Tolga Bayar, Sezer Markirt, Kadir Bıyıklı, Veysi Kavalcı, Mehmet Bozkurt, Mustafa Utkun, Erkan Markirt, Deniz Merde Özdemir, Sabri Abuş

**Affiliations:** 1 Oral and Maxillofacial Surgery, Private Practice, Şırnak, TUR; 2 Cardiology, Adıyaman Training and Research Hospital, Adıyaman, TUR; 3 Cardiology, Koşuyolu High Specialization Education and Research Hospital, İstanbul, TUR; 4 Cardiology, Adıyaman University Faculty of Medicine, Adıyaman, TUR; 5 Cardiology, Kahta State Hospital, Adıyaman, TUR; 6 Oral and Maxillofacial Surgery, Harran University Faculty of Dentistry, Şanlıurfa, TUR

**Keywords:** bruxism, bruxism severity questionnaire, cardiovascular risk, comorbidity, qrisk3

## Abstract

Introduction: Bruxism has been linked to autonomic dysregulation and systemic inflammation-mechanisms that may elevate cardiovascular disease (CVD) risk. However, no study to date has applied a validated risk‐prediction model to quantify this risk in bruxism patients. This study aims to compare 10-year CVD risk, as estimated by the QRISK3 algorithm, between adults with bruxism and matched controls, and to assess the relationship between bruxism severity and cardiovascular risk.

Methods: We conducted a cross‐sectional, matched case-control study recruiting 92 adults (25-65 years) with clinically diagnosed bruxism and 108 non-bruxing controls matched for age (±2 years), sex, body‐mass index (BMI) (±1 kg/m²), smoking status, hypertension, and diabetes. Bruxism severity was measured via the eight-item Bruxism Severity Questionnaire (BSQ). Ten-year CVD risk was calculated using QRISK3 inputs from clinical history, physical examination, and recent laboratory values.

Results: Cases and controls did not differ in mean age (42.5 ± 10.3 vs. 41.8 ± 9.7 years; p=0.63), sex distribution (55% female each), BMI (26.2 ± 3.4 vs. 25.8 ± 3.1 kg/m²; p=0.315), smoking (23.9% vs. 18.5%; p=0.756), hypertension (15.2% vs. 11.1%; p=0.698), or diabetes (8.7% vs. 5.5%; p=0.564). The mean QRISK3 score was higher in bruxers than controls (8.2 ± 4.5 % vs. 6.1 ± 3.7 %; t=4.156, p<0.001), and the prevalence of “high CVD risk” was 28.3 % vs. 14.8 % (χ²=6.102, p=0.013). The BSQ total score correlated moderately with QRISK3 (r=0.36, p<0.001). After adjustment for age, sex, BMI, smoking, hypertension, diabetes, and hyperlipidemia, bruxism remained an independent predictor of high CVD risk (OR 2.30; 95% CI 1.15-4.58; p=0.019).

Conclusion: Adults with bruxism exhibit significantly higher estimated 10-year CVD risk than matched controls, and bruxism severity correlates with QRISK3. These findings support the integration of cardiovascular risk screening in dental practice for patients presenting with bruxism.

## Introduction

Bruxism, defined as involuntary clenching or grinding of the teeth during wakefulness or sleep, is increasingly recognized not only as a stomatognathic disorder but also as a potential harbinger of systemic dysregulation. In particular, sleep bruxism has been linked to heightened sympathetic activity, oxidative stress, and inflammatory marker elevations, all of which are well‐known contributors to cardiovascular disease (CVD) [1].

A growing body of observational evidence supports this connection. For example, a hospital‐based case-control study in India demonstrated a significant association between bruxism and established CVD, suggesting that individuals with bruxism may harbor an elevated burden of cardiovascular risk [2]. Moreover, recent works have documented correlations between the Bruxism Episode Index (BEI) and biomarkers such as C‐reactive protein and fibrinogen, further implicating bruxism in the pathophysiological cascade leading to atherosclerosis and arterial hypertension [3].

Despite these insights, no previous study has employed a comprehensive, validated risk‐prediction tool, such as the QRISK3 algorithm, to quantify the global 10‐year CVD risk in bruxism patients. QRISK3 integrates demographic, clinical, and laboratory parameters to estimate individual CVD risk in populations aged 25-84 and is endorsed by international guidelines for primary prevention stratification.

Accordingly, this case-control study aims to compare QRISK3 scores between adults with clinically confirmed bruxism and matched controls without bruxism, thereby evaluating whether bruxism constitutes an independent marker of elevated cardiovascular risk. Such findings could inform interdisciplinary screening strategies and early preventive interventions for at‐risk dental patients.

## Materials and methods

Study design and participants

A cross-sectional, matched case-control study was conducted at the Cardiology Department of Adıyaman University Faculty of Medicine, Adıyaman, Turkey, between January 2004 and January 2025. Ethical approval for this study was obtained from the Non-interventional Clinical Research Ethics Committee of Adıyaman University, Republic of Turkey (decision date: 24 June 2025; meeting No. 6; decision no. 2025/6-28). Although the study period covered January 2004 to January 2025, this was a retrospective review of existing patient records and clinical data. No interventions or prospective recruitment were performed before ethics approval. The Ethics Committee approval (decision date: 24 June 2025) was obtained prior to initiation of data analysis, in accordance with national regulations for retrospective chart review studies. The study was conducted in accordance with the International Council for Harmonisation’s Good Clinical Practice guidelines, first published in 1996, and the World Medical Association’s Declaration of Helsinki, originally adopted in 1964.

**Figure 1:**
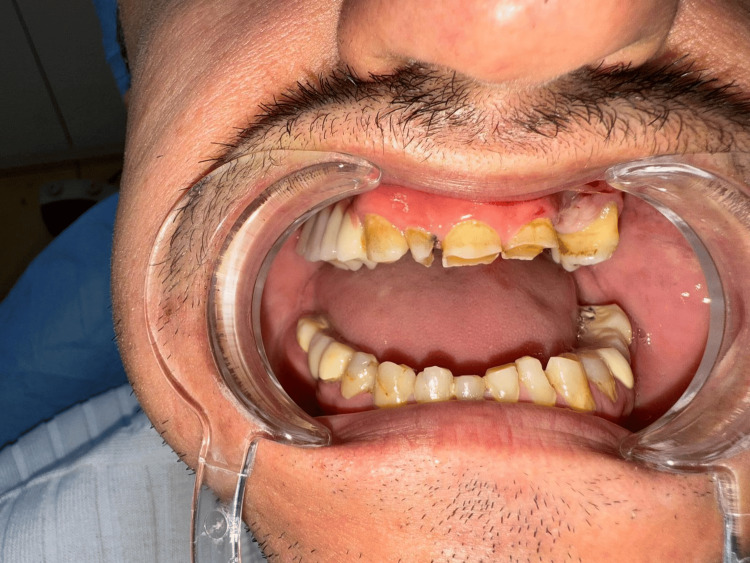
Intraoral clinical photograph showing characteristic enamel wear facets and attrition on the incisal and occlusal surfaces of the maxillary and mandibular anterior teeth, consistent with a diagnosis of bruxism.

**Figure 2:**
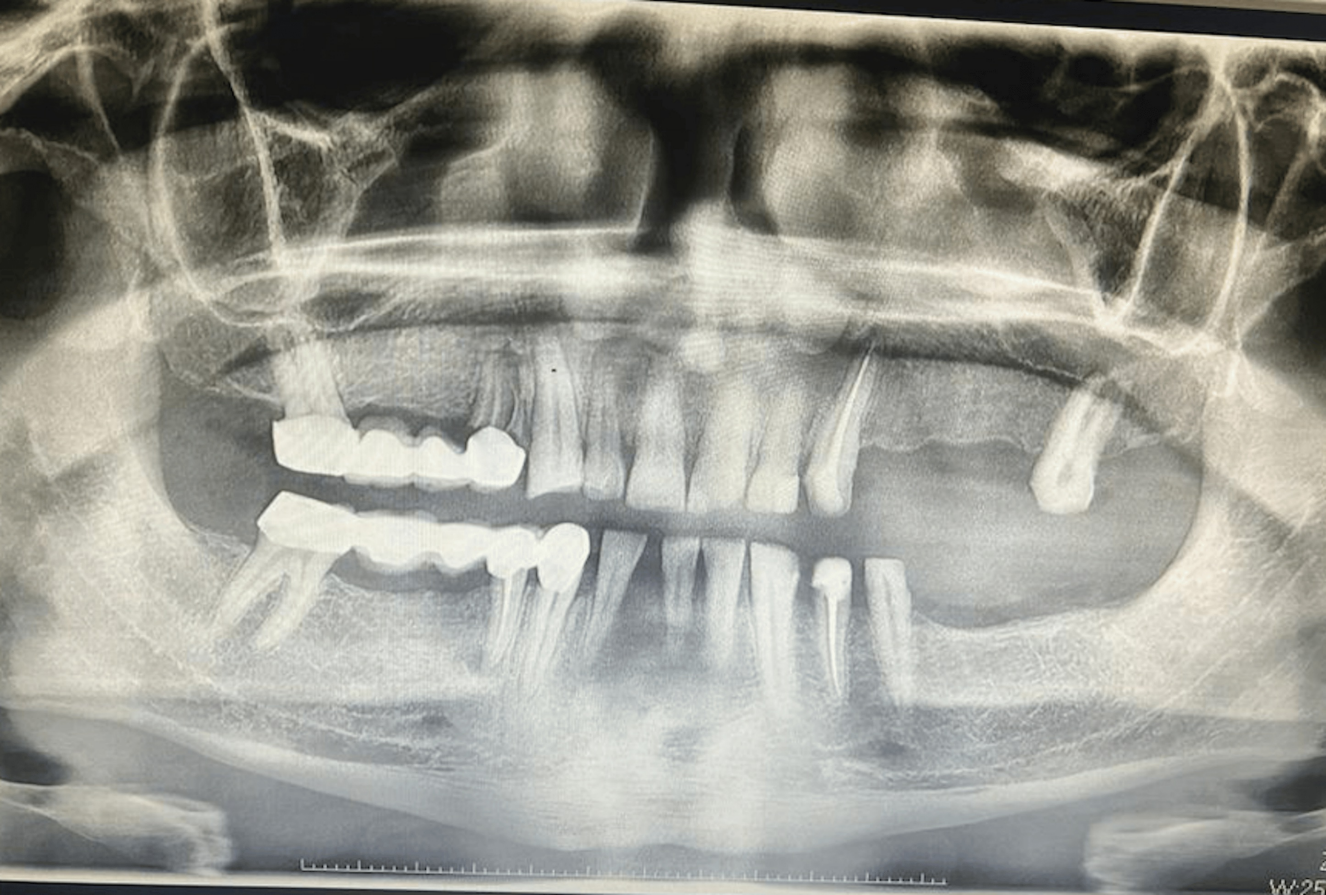
Panoramic radiograph of the same participant, illustrating generalized flattening of the occlusal and incisal surfaces consistent with attrition, as well as overall dental and alveolar bone status without evidence of periapical pathology.

Bruxism diagnosis was based on the gold standard criteria recommended by the International Classification of Sleep Disorders, 3rd edition [4], and the recent International Consensus on Bruxism [5]. Specifically, diagnosis required (i) self-reported tooth clenching or grinding during wakefulness or sleep and (ii) at least one clinical sign on examination by an oral and maxillofacial surgeon (e.g., enamel wear facets, incisal/occlusal attrition, or masticatory muscle tenderness) [5]. The study included 92 adults with clinically diagnosed bruxism (cases). For each case, a control participant without any history of bruxism was selected. Controls (n=108) were matched to cases by age (±2 years), sex, body mass index (BMI, ±1 kg/m²), smoking status, presence of hypertension, and diabetes mellitus. All participants were between 25 and 65 years of age. Exclusion criteria were stage 4-5 chronic kidney disease, active malignancy, chronic inflammatory or autoimmune disorders, and current use of psychiatric medications. Participants with diagnosed obstructive sleep apnea (OSA) or those under treatment for OSA (e.g., continuous positive airway pressure (CPAP) therapy, mandibular advancement devices) were excluded to avoid confounding effects on cardiovascular risk. The intraoral photo of one participant is presented in Figure 1, and the corresponding panoramic image is shown in Figure 2.

Bruxism assessment

Bruxism severity was quantified using the eight-item Bruxism Severity Questionnaire (BSQ) (Table 1). Each item was scored 0-4, yielding a total score range of 0-32. Internal consistency in our sample was excellent (Cronbach’s α = 0.88). A single trained examiner administered the BSQ in a quiet room immediately prior to any other clinical measurements. The BSQ was specifically designed for this study based on the methodologies of prior investigations [5,6]. Severity was quantified using the BSQ. A BSQ score ≥8 was considered indicative of clinically relevant bruxism, and scores were further categorized as mild (8-15), moderate (16-23), and severe (≥24).

**Table 1:** Bruxism Severity Questionnaire (BSQ) Items 0: never; 1: rarely; 2: occasionally; 3: often; 4: very often

Item	Question	Response Options (0–4)
1	How often do you clench your teeth during the day?	
2	How often do you grind your teeth during the day?	
3	How often do you clench your teeth during sleep (as far as you know)?	
4	How often do you grind your teeth during sleep (as far as you know)?	
5	How often do you wake up with jaw pain or soreness?	
6	How often do you have headaches on waking?	
7	How often do you notice wear facets (flat shiny spots) on your teeth?	
8	Overall, how severe would you rate your teeth‐grinding/‐clenching problem?	

Cardiovascular risk estimation

Ten-year cardiovascular risk was estimated for each participant using the QRISK3 algorithm (version 2023, developed by Professor Julia Hippisley-Cox and Dr. Carol Coupland at the University of Nottingham, Nottingham, UK, using data from the QResearch database), which integrates demographic, clinical, and laboratory parameters (Appendix A). Required inputs (age, sex, ethnicity, systolic blood pressure, total/HDL cholesterol, smoking status, diabetes, chronic kidney disease, atrial fibrillation, migraine, corticosteroid use, systemic lupus erythematosus, severe mental illness, atypical antipsychotic use, erectile dysfunction, blood pressure treatment, BMI, and family history of premature coronary heart disease) were collected from patient charts or measured at the time of enrollment [7].

Data collection

Data collection encompassed a comprehensive set of variables beyond the BSQ and QRISK3 inputs. Demographic information, including age, sex, height, and weight (used to calculate BMI), was recorded for each participant. Lifestyle factors were documented by noting current smoking status (yes/no), average weekly alcohol intake (in standard units), and self-reported hours of physical activity per week. Medical history was obtained with particular attention to the presence of hypertension, diabetes mellitus, hyperlipidemia, and a family history of premature CVD. Finally, laboratory results from within the previous six months, like total cholesterol, high-density lipoprotein (HDL) cholesterol, low-density lipoprotein (LDL) cholesterol, estimated glomerular filtration rate (eGFR), and systolic blood pressure, were extracted from medical records to complete the dataset.

Statistical analysis

Statistical analyses were conducted using IBM SPSS Statistics software, version 28.0 (IBM Corp., Armonk, NY). Continuous variables were first evaluated for normality with the Shapiro-Wilk test. Variables that were normally distributed are reported as mean ± standard deviation and were compared between groups using independent-samples t-tests; variables that deviated from normality are presented as median (interquartile range) and were compared using the Mann-Whitney U test. Categorical variables are expressed as counts and percentages and were compared with either the chi-square test or Fisher’s exact test, as appropriate. To assess the association between bruxism severity and cardiovascular risk, Pearson’s correlation coefficient (r) was calculated between the BSQ total score and QRISK3 score. For multivariable analysis, a logistic regression model was constructed with high CVD risk (QRISK3 ≥ 10%) as the dependent outcome. Independent predictors included bruxism status (yes/no), age (per 10-year increment), sex, BMI (per 1 kg/m²), current smoking, hypertension, diabetes mellitus, and hyperlipidemia. Adjusted odds ratios (ORs) with 95% confidence intervals (CI) are reported. All statistical tests were two-tailed, and a p-value < 0.05 was considered statistically significant. Continuous predictors in the regression were scaled to clinically meaningful units (10-year increases for age and 1 kg/m² increments for BMI). p<0.05 is accepted as a statistically significant value.

## Results

Results

A total of 200 participants were included (92 cases with bruxism, 108 matched controls).

Sociodemographic and comorbidity profile

The mean age of the bruxism group was 42.5 ± 10.3 years versus 41.8 ± 9.7 years in controls (t = 0.484, p = 0.634). Both groups were 55% female. Mean BMI was 26.2 ± 3.4 kg/m² in bruxism cases and 25.8 ± 3.1 kg/m² in controls (t = 1.020, p = 0.315). Current smoking prevalence was 23.9% versus 18.5% (χ² = 0.104, p = 0.756). Rates of hypertension (15.2% vs 11.1%, p = 0.698), diabetes mellitus (8.7% vs 5.5%, p = 0.564), and hyperlipidemia (19.6% vs 14.8%, p = 0.746) did not differ significantly. A positive family history of CVD was reported by 29.3% of cases and 27.7% of controls (p = 0.786) (Table 2).

**Table 2:** Sociodemographic and comorbidity comparison between bruxism cases and controls The data have been represented as N (%) or Mean±SD. ^1^Student's t-test was used. ^2^Chi-square test was used. p<0.05 was accepted as statistical significance. CVD: cardiovascular disease

Variable	Bruxism (n=92)	Control (n=108)	Test Statistics	p-value
Age, mean ± SD (years)	42.5 ± 10.3	41.8 ± 9.7	t = 0.484	0.6341
Female sex, n (%)	51 (55.4%)	60 (55.5%)	χ² = 0.001	1.0002
BMI, mean ± SD (kg/m²)	26.2 ± 3.4	25.8 ± 3.1	t = 1.020	0.3151
Current smokers, n (%)	22 (23.9%)	20 (18.5%)	χ² = 0.104	0.7562
Hypertension, n (%)	14 (15.2%)	12 (11.1%)	χ² = 0.162	0.6982
Diabetes mellitus, n (%)	8 (8.7%)	6 (5.5%)	χ² = 0.344	0.5642
Hyperlipidemia, n (%)	18 (19.6%)	16 (14.8%)	χ² = 0.112	0.7462
Family CVD history, n (%)	27 (29.3%)	30 (27.7%)	χ² = 0.087	0.7862

QRISK3 scores

The mean QRISK3 score was significantly higher in the bruxism group (8.2 ± 4.5%) compared with controls (6.1 ± 3.7%) (t = 4.156, p < 0.001). The proportion of participants categorized as high CVD risk (QRISK3 ≥ 10%) was 28.3% in bruxism cases versus 14.8% in controls (χ² = 6.102, p = 0.013) (Table 3).

**Table 3:** QRISK3 Score outcomes in cases vs. controls The data have been represented as N (%) or Mean±SD. ^1^Student's t-test was used. ^2^Chi-square test was used. p<0.05 was accepted as statistical significance. CVD: cardiovascular disease

QRISK3 Metric	Bruxism (n=92)	Control (n=108)	Test Statistics	p-value
QRISK3 score, mean ± SD (%)	8.2 ± 4.5	6.1 ± 3.7	t = 4.156	<0.001^1^
High CVD risk (≥10%), n (%)	26 (28.3%)	16 (14.8%)	χ² = 6.102	0.0132

Correlation between bruxism severity and CVD risk

Pearson's correlation analysis demonstrated a moderate positive association between BSQ total score and QRISK3 score (r = 0.364, 95% CI 0.212-0.498; p < 0.001) (Table 4).

**Table 4:** Correlation Between BSQ score and QRISK3 score Pearson's correlation test was used. p<0.05 was accepted as statistical significance. BSQ: Bruxism Severity Questionnaire

Measure	Pearson's r	95% CI	p-value
BSQ total score vs. QRISK3	0.364	0.212 – 0.498	<0.001

Multivariable logistic regression

After adjusting for age, sex, BMI, smoking, hypertension, diabetes, and hyperlipidemia, bruxism remained an independent predictor of high CVD risk (OR 2.30, 95% CI 1.15-4.58; p = 0.019). Other significant predictors included age (per 10-year increase: OR 1.45, 95% CI 1.10-1.91; p = 0.008), BMI (per 1 kg/m²: OR 1.08, 95% CI 1.01-1.16; p = 0.027), and hypertension (OR 3.50, 95% CI 1.52-8.05; p = 0.003). Female sex, current smoking, diabetes, and hyperlipidemia did not reach statistical significance in the adjusted model (Table 5).

**Table 5:** Multivariable logistic regression for high CVD risk (QRISK3 ≥10%) Multivariable logistic regression analysis test was used. p<0.05 was accepted as statistical significance. CVD: cardiovascular disease

Predictor	OR (95% CI)	p-value
Bruxism (yes vs. no)	2.30 (1.15 – 4.58)	0.019
Age (per 10-year increase)	1.45 (1.10 – 1.91)	0.008
Female sex	0.85 (0.42 – 1.70)	0.644
BMI (per 1 kg/m²)	1.08 (1.01 – 1.16)	0.027
Current smoking	1.75 (0.82 – 3.75)	0.156
Hypertension	3.50 (1.52 – 8.05)	0.003
Diabetes mellitus	2.10 (0.72 – 6.11)	0.175
Hyperlipidemia	1.90 (0.88 – 4.12)	0.102

## Discussion

In this case-control study, we demonstrated that adults with bruxism exhibit significantly higher QRISK3-estimated 10-year CVD risk compared with matched controls. Our findings, mean QRISK3 scores of 8.2% in bruxers versus 6.1% in controls (p < 0.001) and a two-fold increase in odds of “high CVD risk” (QRISK3 ≥ 10%) after multivariable adjustment, support the hypothesis that bruxism may serve as an independent marker of elevated cardiovascular risk. Below, we place these results in the context of existing literature, explore potential mechanisms, consider clinical implications, and identify directions for future research.

Although bruxism is primarily viewed as an oromandibular parafunctional activity, a growing body of evidence links sleep bruxism with adverse cardiovascular endpoints. A recent systematic review found associations between sleep bruxism and hypertension, arrhythmias, and ischemic heart disease, positing that chronic sympathetic overactivity and oxidative stress may underlie these links [3]. Polysomnographic studies have shown that rhythmic masticatory muscle activity in bruxers is temporally coupled with transient surges in blood pressure and heart rate, analogous to arousals in OSA [8]. In normotensive subjects, higher BEI correlates with elevated ambulatory blood pressure readings, further implicating bruxism in early hemodynamic dysregulation [9].

In parallel, biochemical investigations reveal that bruxism intensity correlates positively with circulating inflammatory markers, including C-reactive protein (CRP) and fibrinogen, which are well-established mediators of endothelial dysfunction and atherogenesis [10,11]. Elevated oxidative stress markers in bruxers further reinforce the concept that repetitive masticatory muscle contractions may trigger systemic inflammatory cascades, promoting vascular injury [12]. Together, these pathophysiological pathways converge on established QRISK3 components, namely hypertension, dyslipidemia, and inflammatory milieu, providing mechanistic plausibility to our observed association.

To our knowledge, no prior investigation has directly applied a comprehensive CVD risk calculator to bruxism patients. However, case-control analyses using surrogate outcomes have yielded similar signals. A hospital‐based study in India reported that patients with sleep bruxism had a higher prevalence of diagnosed hypertension and diabetes compared with non-bruxers (OR 1.9 and 1.7, respectively) after controlling for age and BMI (2). In an observational study by Marconcini et al., cardiac patients demonstrated a 91.7% prevalence of bruxism compared with 28.3% in non‐cardiac controls, corresponding to an adjusted OR of 3.24 for bruxism among those with CVD (p<0.01) [13]. These studies complement our findings by highlighting that bruxism correlates not only with risk factors but also with subclinical endovascular damage.

Our correlation analysis (r = 0.364 between BSQ and QRISK3) aligns with prior work showing moderate associations (r ≈ 0.30-0.40) between bruxism severity and CRP, fibrinogen, or ambulatory blood pressure fluctuations [9,10]. Collectively, these data support a dose-response relationship: more severe bruxism correlates with higher systemic risk marker levels, which likely translates into higher aggregate CVD risk via QRISK3 computation.

Recent evidence also suggests that individual chronotype may modulate the bruxism-cardiovascular risk relationship. An eveningness chronotype has been identified as a risk factor for awake bruxism, potentially reflecting underlying circadian dysregulation of autonomic tone and stress response pathways [14]. Incorporating chronotype assessment into future studies could therefore enhance risk stratification by capturing the influence of internal circadian timing on both oromandibular muscle activity and cardiovascular function.

Moreover, the model of sleep bruxism proposed by Lavigne et al. [15] highlights that rhythmic masticatory muscle activity is intimately linked to brief autonomic-cardiac and brainstem arousal events, suggesting that each grinding episode may be triggered by transient sympathetic surges and micro-arousals preceding jaw muscle activation. Integrating these insights into our framework implies that the very mechanisms driving sleep bruxism, namely, autonomic-motor coupling and micro-arousal dynamics, may also precipitate the repeated hemodynamic fluctuations that cumulatively increase cardiovascular risk in this population.

Additionally, the trigeminal cardiac reflex (TCR) model posits that mandibular muscle activation during sleep bruxism triggers a potent autonomic reflex arc, causing abrupt bradycardia and transient hypotension each time the trigeminal nerve is stimulated [16]. Over the long term, these repetitive TCR‐mediated cardiovascular oscillations may contribute to endothelial shear stress and maladaptive vascular remodeling, further compounding the chronic risk elevation captured by QRISK3 [17].

A recent polysomnographic investigation by Nukazawa et al. [18] demonstrated that sleep bruxism events follow a very consistent autonomic sequence: sympathetic activation, then EMG-defined masticatory bursts, and finally parasympathetic rebound, in over 90% of occurrences. This rhythmic autonomic-motor coupling mirrors the patterns we propose underlie long-term vascular stress: each bruxism episode could act like a mini‐hemodynamic insult, repeatedly subjecting the endothelium to shear and pressure fluctuations that promote arterial remodeling and hypertension. Incorporating continuous electrocardiogram (ECG) and electromyography (EMG) monitoring into future longitudinal cohorts would therefore be invaluable for directly linking these acute autonomic oscillations to the progressive cardiovascular risk accrual captured by QRISK3.

Several interrelated mechanisms may explain the observed association between bruxism and elevated cardiovascular risk. First, autonomic dysregulation appears to play a central role. Bruxism episodes are often accompanied by transient surges in sympathetic nervous system activity and withdrawal of parasympathetic (vagal) tone, resulting in episodic elevations in blood pressure and heart rate. Over time, these repetitive hemodynamic stresses may lead to structural changes in the vasculature and contribute to the development of hypertension, independent of traditional risk factors [8]. Second, bruxism is associated with a proinflammatory and pro-oxidative milieu. Patients who grind their teeth demonstrate higher circulating levels of C-reactive protein and fibrinogen, indicating a chronic low-grade inflammatory state. Concurrently, markers of oxidative stress, such as increased malondialdehyde and altered thiol status, have been documented, suggesting that lipid peroxidation and endothelial injury pathways are amplified in bruxism, thus promoting atherosclerosis [12]. Third, neuroendocrine activation may further exacerbate cardiovascular risk. Stress-related hormones, including cortisol and catecholamines, are elevated in individuals with bruxism, and urinary 17-hydroxycorticosteroid levels correlate with bruxism episode indices. This hypercortisolemic state can worsen insulin resistance, dyslipidemia, and visceral fat accumulation, components explicitly captured by the QRISK3 algorithm [19]. Together, these mechanisms, autonomic dysregulation, systemic inflammation with oxidative stress, and neuroendocrine activation, likely act in concert to accelerate vascular remodeling and increase long-term cardiovascular risk. Importantly, these effects may begin manifesting even in younger, normotensive patients who exhibit bruxism, highlighting the importance of early screening and intervention.

Our data suggest that dental practitioners identifying bruxism might consider referral for cardiovascular risk assessment. Incorporating QRISK3 into dental clinics (via brief questionnaire and basic vitals) could flag high‐risk individuals for primary care or cardiology evaluation. Early identification provides an opportunity to implement lifestyle interventions (diet, exercise, sleep hygiene) and pharmacotherapy (antihypertensives, statins) before overt CVD emerges.

Furthermore, interdisciplinary collaboration between dentists, sleep specialists, and cardiologists may optimize both bruxism management and CVD prevention. For instance, botulinum toxin injections reduce bruxism severity and might attenuate associated blood pressure surges, a potential dual benefit that requires further study [20].

Strengths of our study include a matched case-control design controlling for major QRISK3 inputs, use of a validated bruxism severity questionnaire (BSQ; Cronbach’s α = 0.88), and application of a robust, guideline-endorsed risk algorithm (QRISK3).

Limitations of this study should be acknowledged. First, the cross-sectional design precludes any causal inference; although we observed significant associations between bruxism and QRISK3-estimated cardiovascular risk, temporality and directionality cannot be established. Longitudinal or interventional studies are needed to clarify whether bruxism contributes to the development of CVD or merely coexists with other risk factors. Second, we relied on QRISK3 scores as surrogate outcomes rather than documented cardiovascular events such as myocardial infarction or stroke. While QRISK3 is a validated tool, risk prediction models may overestimate or underestimate risk in certain populations, particularly outside the UK setting where the algorithm was originally derived. Third, despite controlling for major confounders such as age, sex, BMI, hypertension, diabetes, and smoking, residual confounding cannot be excluded. Important unmeasured variables include severity of obstructive sleep apnea, psychological stress levels, sleep quality, dietary habits, and socioeconomic status, all of which may independently influence cardiovascular risk and bruxism expression. Fourth, the diagnosis of bruxism was based on self-report and clinical examination supplemented by the BSQ. Although this approach is widely used in epidemiological research, it does not achieve the diagnostic precision of polysomnography (PSG) or electromyographic recordings, which remain the gold standard. Fifth, the study was conducted in a single-center setting in Turkey, which may limit the generalizability of findings to other ethnic and geographic populations. Sixth, potential recall bias related to self-reported bruxism and lifestyle factors (e.g., smoking, physical activity, alcohol consumption) cannot be excluded. Finally, sample size, although adequate for detecting differences in QRISK3, may not have been sufficient to explore subgroup analyses (e.g., sex-specific associations or dose-response relationships).

Future research should pursue several key avenues to deepen our understanding of the bruxism-CVD connection and translate these insights into clinical practice. First, prospective cohort studies that longitudinally track changes in bruxism severity alongside QRISK3 scores and the occurrence of hard CVD endpoints, such as myocardial infarction and stroke, will be critical for establishing temporal and causal relationships. Second, interventional trials are needed to determine whether therapies specifically targeting bruxism (for example, occlusal splints, botulinum toxin injections, or structured stress‐management programs) can lower QRISK3 scores or favorably modify circulating CVD biomarkers. Third, mechanistic investigations should employ detailed assessments of endothelial function (for instance, flow‐mediated dilation), measures of arterial stiffness (pulse wave velocity), and autonomic parameters (heart‐rate variability) to clarify the biological pathways that link repetitive masticatory muscle activity to vascular injury. Finally, if bruxism proves to be an independent predictor of CVD risk beyond traditional QRISK3 variables, future risk‐prediction models could be enhanced by formally incorporating bruxism severity as a novel risk factor, thereby improving their ability to identify at‐risk individuals and guide early preventive interventions.

## Conclusions

This study represents the first application of QRISK3 to quantify cardiovascular risk in adults with bruxism, revealing a significant and independent association between bruxism and elevated predicted CVD risk. These findings underscore the value of interdisciplinary risk screening and suggest that bruxism may serve as an early marker of systemic vascular dysfunction. Integrating bruxism assessment into routine risk stratification protocols could enable timely preventive interventions, thereby mitigating long-term cardiovascular morbidity in this at‐risk population.

## Figures and Tables

**Figure 3:**
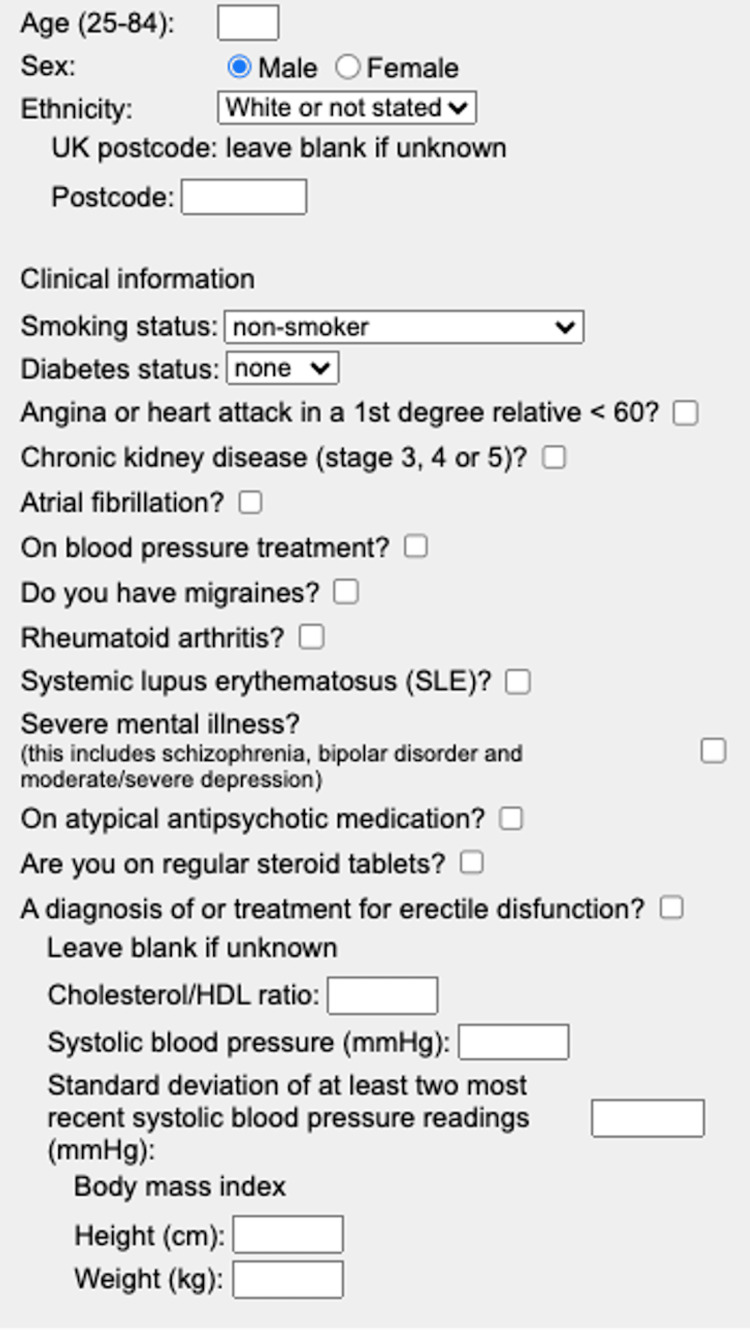
Screenshot of the QRISK3 online calculator interface, displaying input fields for key demographic (age, sex, ethnicity, postcode), lifestyle (smoking status), clinical (diabetes, hypertension treatment, atrial fibrillation, chronic kidney disease, family history of premature cardiovascular disease (CVD), rheumatoid arthritis, lupus, migraine, mental illness, steroid use, erectile dysfunction), laboratory (cholesterol/high-density lipoprotein (HDL) ratio, systolic blood pressure, blood pressure variability), and anthropometric (height, weight, BMI) variables required to compute the 10-year cardiovascular risk score. Source: [7]

## Appendices

Appendix A 

## References

[1] Atilgan Z, Buyukkaya R, Yaman F (2011). Bruxism: is it a new sign of the cardiovascular diseases?. Eur Rev Med Pharmacol Sci.

[2] Bhatia R, H. DN, Bhatnagar MK, Sood S, Kumar P, Chaudhary R (2021). A hospital based case control study to explore the association between bruxism and cardiovascular diseases in Kangra region of Himachal Pradesh. Int J Community Med Public Health.

[3] Michalek-Zrabkowska M, Martynowicz H, Wieckiewicz M, Smardz J, Poreba R, Mazur G (2021). Cardiovascular implications of sleep bruxism-a systematic review with narrative summary and future perspectives. J Clin Med.

[4] Sateia MJ (2014). International classification of sleep disorders-third edition: highlights and modifications. Chest.

[5] Verhoeff MC, Lobbezoo F, Ahlberg J (2025). Updating the bruxism definitions: report of an international consensus meeting. J Oral Rehabil.

[6] Winocur E, Uziel N, Lisha T, Goldsmith C, Eli I (2011). Self-reported bruxism - associations with perceived stress, motivation for control, dental anxiety and gagging. J Oral Rehabil.

[7] (2025). QRISK®3 risk calculator. https://www.qrisk.org/.

[8] Nashed A, Lanfranchi P, Rompré P (2012). Sleep bruxism is associated with a rise in arterial blood pressure. Sleep.

[9] Michalek-Zrabkowska M, Wieckiewicz M, Gac P (2021). Effect of sleep bruxism intensity on blood pressure in normotensives. J Clin Med.

[10] Michalek-Zrabkowska M, Wieckiewicz M, Smardz J (2020). Determination of inflammatory markers, hormonal disturbances, and sleepiness associated with sleep bruxism among adults. Nat Sci Sleep.

[11] Chandy S, Joseph K, Sankaranarayanan A, Issac A, Babu G, Wilson B, Joseph J (2017). Evaluation of C-reactive protein and fibrinogen in patients with chronic and aggressive periodontitis: a clinico-biochemical study. J Clin Diagn Res.

[12] Fulek M, Frosztega W, Wieckiewicz M (2025). The link between sleep bruxism and oxidative stress based on a polysomnographic study. Sci Rep.

[13] Marconcini S, Giammarinaro E, Cosola S (2018). Bruxism and cardiovascular diseases: a cross-sectional study. J Cardiol Ther.

[14] Meira E Cruz M, Winocur E, Gozal D, Lavigne GJ (2021). Chronotype and bruxism: should we look further and get it from the heart?. Cranio.

[15] Lavigne GJ, Huynh N, Kato T, Okura K, Adachi K, Yao D, Sessle B (2007). Genesis of sleep bruxism: motor and autonomic-cardiac interactions. Arch Oral Biol.

[16] Schames SE, Schames J, Schames M, Chagall-Gungur SS (2012). Sleep bruxism, an autonomic self-regulating response by triggering the trigeminal cardiac reflex. J Calif Dent Assoc.

[17] Chatzizisis YS, Coskun AU, Jonas M, Edelman ER, Feldman CL, Stone PH (2007). Role of endothelial shear stress in the natural history of coronary atherosclerosis and vascular remodeling: molecular, cellular, and vascular behavior. J Am Coll Cardiol.

[18] Nukazawa S, Yoshimi H, Sato S (2018). Autonomic nervous activities associated with bruxism events during sleep. Cranio.

[19] Martynowicz H, Wieckiewicz M, Poreba R (2019). The relationship between sleep bruxism intensity and renalase concentration-an enzyme involved in hypertension development. J Clin Med.

[20] Coelho MS, Oliveira JM, Polmann H, Pauletto P, Stefani CM, Maciel LC, Canto GL (2025). Botulinum toxin for bruxism: an overview. Toxins (Basel).

